# 3D Macroporous Zinc Compound/Silicone Hybrid Foams for Amperometric Sensing of Glucose Oxidase

**DOI:** 10.1002/gch2.201800049

**Published:** 2018-11-25

**Authors:** Ye Wu, Hao Fu, Weiwei Xie, Yingcheng Lin, Orhan Kizilkaya, Jian Xu

**Affiliations:** ^1^ Division of Electrical and Computer Engineering Louisiana State University Baton Rouge LA 70803 USA; ^2^ Department of Mechanical Engineering Mcgill University 817 Sherbrooke St. West Montreal Quebec H3A 0C3 Canada; ^3^ Department of Chemistry Louisiana State University Baton Rouge LA 70803 USA; ^4^ Key Laboratory of Dependable Service Computing in Cyber Physical Society (Chongqing University) of Ministry of Education Chongqing 400044 China; ^5^ College of Communication Engineering Chongqing University Chongqing 400044 China; ^6^ Center for Advanced Microstructures and Devices Louisiana State University 6980 Jefferson Hwy. Baton Rouge LA 70806 USA

**Keywords:** glucose oxidase sensing, metal organic materials, XANES, zinc compounds, zinc oxide

## Abstract

A 3D porous matrix makes an intriguing sensing platform, which can integrate functional guest molecules. Here, the first demonstration of a zinc compound/silicone hybrid foam is reported for amperometric sensing of glucose oxidase. The silicone foam is fabricated by a self‐developed solid‐filling‐melting method. Two zinc‐based polymers, Zn‐Compound‐1 and Zn‐Compound‐2, are synthesized. Zn‐Compound‐1 and Zn‐Compound‐2 are characterized by X‐ray diffraction, X‐ray photoelectron spectroscopy, Fourier‐transform infrared spectroscopy, Raman spectroscopy, and X‐ray absorption nearedge structure spectroscopy of carbon K‐edge, oxygen K‐edge, and zinc L‐edge. Effective amperometric sensing of glucose oxidase is achieved by introducing Zn‐Compound‐1 or Zn‐Compound‐2 into the silicone foam, i.e., an increase of the concentration of the glucose oxidase led to an increase of detected current. This phenomenon can be explained by a possible mechanism of the formation of electron extra bands.

## 1 Introduction

Research interest in seeking simple, affordable and portable biochemical sensing platforms is growing, which is especially useful in developing countries, resource‐limited regions, and rural areas.^1^, ^2^, ^3^

Following this trend, polymer foams based platform can be one of the answers to this demand. The polymer foams are a new class of porous materials made in a way through the generation of arbitrary holes inside a silicone bulk. The generation of porous structure can be fulfilled through template melting, gas filling, 3D printing, etc.^4^ The porous structure of the polymer foam provides it with a flexible matrix for introducing guest molecules, which makes it useful as functional material, such as flexible conductors^5^, ^6^, ^7^ and photocatalysts.^8^

The metal compounds are a new class of crystals made through the combination of inorganic metal ions and organic molecules.^9^ The metal (e.g., Zn^2+^) compounds are widely used in various chemical applications. For instance, Zn_4_O(1,4‐benzenedicarboxylate)_3_ was used for hydrogen storage^10^; Zn(1,4‐benzenedicarboxylic acid)(4,4′‐bipyridine)_0.5_ for alkanes separation,^11^ {[Zn(L1)]*_x_*H_2_O*_y_*DMF}*_n_* (L1 = 2‐methoxyimidazolate‐4‐amide‐5‐imidate, DMF = *N*,*N*‐dimethylformamide) for carbon dioxide capture,^12^ and {[Zn(H_2_O)(C_5_H_7_NO_4_)]H_2_O}*_n_* for catalysis.^13^ Strengthening effort is being put forth to investigate the potential application of the zinc compounds as a sensing platform. Several examples can be found: 1) {[Zn(HCbdcp)_2_]H_2_O}*_n_* (Cbdcp = *N*‐(4‐carboxybenzyl)‐(3,5‐dicarboxyl)pyridinium) was used for sensing of human immunodeficiency virus‐1 ds‐DNA sequences;^14^ 2) [Zn(PAM)(en)] (PAM = 4,4′‐methylenebis(3‐hydroxy‐2‐naphthalenecarboxylate), en = 1,2‐ethanediamine) for sensing of 2,4,6‐trinitrophenol and Cu^2+^ ion;^15^ 3) two zinc(II) coordination polymers [Zn_2_(tib)_2_(H_2_BDC‐Br = 2‐bromo‐1,4‐benzenedicarboxylic acid)]_2_·_2_SO_4_·17H_2_O and [Zn_4_(tib)_2_(BDC‐Br)_3_(H_2_O)_4_SO_4_]·7.5H_2_O·2.5DMF (tib = 1,3,5‐tris(1‐imidazolyl)benzene) for sensing of acetone;^16^ Zn_3_(H_3_BTC = benzen‐1,3,5‐tricarboxylic acid)_2_·12H_2_O for sensing of organoamines;^17^ [Zn_2_(TPOM)(NDC)_2_]·3.5H_2_O (TPOM = tetrakis(4‐pyridyloxymethylene)methane, H_2_ndc = 2,6‐naphthalenedicarboxylic acid) for sensing of Fe(III) and Cr(VI) Ions;^18^ [Zn_7_O_2_(bpdc)_4_(dmpp)_2_]·6DEF·10H_2_O (H_2_bpdc = 4,4′‐biphenyldicarboxylic acid, Hdmpp = 3,5‐dimethyl‐4‐(4′‐pyridyl)pyrazole) for sensing of nitrobenzene molecule over toluene, *p*‐xylene, mesitylene, and cyclohexane;^19^ given that they hold the advantages of easy synthesis, flexible structure, and the combination of both organic and inorganic components.^9^, ^10^, ^11^, ^12^, ^13^, ^14^, ^15^, ^16^, ^17^, ^18^, ^19^

Glucose oxidase (Gox), a model enzyme in biochemistry, is generally used with an oxygen electrode for glucose monitoring. Gox‐based analytical devices are extensively studied because assessing glucose levels is among the most important analytical tasks and glucose test is a common task in all blood tests and biosensors.^20^ Gox is also widely used for investigation of new immobilization systems.^20^ However, in case of Gox‐based analytical devices and immobilization methods the final concentration of enzyme is not known.^20^ Therefore, it is necessary to develop facile method for assessing the amount of enzyme present in the samples. Gox can be detected through electrochemical station with CdS nanoparticles modified electrode.^21^ Additionally, direct electrochemistry between Gox and carbon nanotubes was reported.^22^ These works may propose the sensing of Gox through impedance spectroscopy or electrochemical station with the nanomaterial‐based electrodes.

It should be noted the zinc compounds or CdS or carbon nanomaterials based sensors reported are performed via using luminescent/fluorescence/Raman/impedance spectroscopy or electrochemical station,^9^, ^10^, ^11^, ^12^, ^13^, ^14^, ^15^, ^16^, ^17^, ^18^, ^19^, ^21^, ^22^ which involved bulky and sophisticated instruments. In addition, there are no reports on amperometric application of the metal compounds as a sensing materials via simple electrical current measurement.

Here, we report on directly sensing Gox by Zn compound/silicone hybrid foam. Two kinds of zinc‐based metal compounds were prepared, Zn‐Compound‐1 and Zn‐Compound‐2. Silicone foam was fabricated by a self‐developed solid‐filling‐melting technology and filled with Zn‐Compound‐1 or Zn‐Compound‐2. The synthesized Zn‐Compound‐1 or Zn‐Compound‐2 was characterized by powder X‐ray diffraction (XRD), X‐ray photoelectron spectroscopy (XPS), Fourier‐transform infrared spectroscopy (FTIR), Raman spectroscopy and X‐ray absorption near edge structure spectroscopy (XANES) of carbon K‐edge, oxygen K‐edge, and zinc L‐edge.

We studied the electrical current generated from the introduction of Gox to the Zn‐compound/silicone hybrid foam. We found the magnitude of the electrical current was dependent on the concentration of Gox. This behavior was well explained by a possible mechanism of extra bands formation. Due to the facile amperometric detection and its exceptional performance, the Zn‐compound/silicone hybrid foam is envisioned to play significant roles in many low‐cost, rapidly processed, and portable biochemical‐sensing applications.

## 2 Results and Discussion

The synthesis of Zn‐Compound‐1 and Zn‐Compound‐2 was done in a facile chemistry (see the Experimental Section). The simulated XRD profile was in good agreement with the experimental (see Figure S1, Supporting Information). The crystal cell parameters of Zn‐Compound‐1 and Zn‐Compound‐2 were shown in Table S1 in Supporting Information. After the synthesis of the Zn‐Compound‐1 and Zn‐Compound‐2, silicone foam was made and used as a matrix to host them.

**Figure 1:**
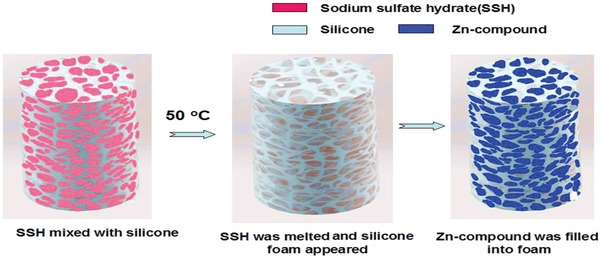
Schematic of the fabrication of Zn compound/silicone hybrid foam.

The silicone foam was fabricated via a self‐developed solid‐filling‐melting technology (**Figure** **1** illustrated the fabrication schematic). Initially, a cup of silicone gel was formed by mixing and stirring silicone gel (2 g) and sodium sulfate hydrate (8 g). It should be mentioned that we had to use a Teflon rod (length = 10 cm) driven by a DC motor with rated torque 21 kg cm to stir the silicone gel. Because the silicone gel was very sticky and ordinary magnetic stirrer was not workable.

After 0.5 h, the composite gel silicone/sodium sulfate hydrate was solidified. It was heated on a hotplate with a temperature of 50–60 ˚C. After the sodium sulfate hydrate was melted, porous structure of silicone bulk would appear. The porous silicone bulk was stirred and washed in ultrapure water for 1 h to clean out the residue of sodium sulfate hydrate. Then it was squeezed several times to wipe out the water. The acquired silicone foam was dried completely via heating (50–60 °C) for 2 h. Then it was cut into rectangle shape (length: 1.5 cm, width: 1.2 cm, and depth: 0.8 cm).

**Figure 2:**
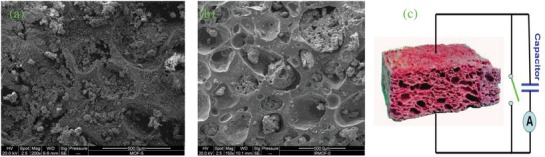
a) SEM image of silicone foam filled with Zn‐Compound‐1 crystals. b) SEM image of silicone foam filled with Zn‐Compound‐2 crystals. c) Schematic of measurement setup for sensing glucose oxidase with different concentration. A fabricated silicone foam was filled with Zn‐compound. After glucose oxidase was injected into the foam by a syringe, current would be generated and stored in a capacitor. When the switch turned closed, the capacitor discharged through a microampere meter.

The synthesized Zn‐compound powder in ethanol solution was filled into the silicone foam by a pipette. The scanning electron microscope (SEM) images confirmed that the sample powders were filled in the holes of the silicone foam (**Figure** **2**a,b).

Soft X‐ray absorption spectroscopy near edge structure (XANES) measurements of the carbon and oxygen K‐edge and zinc L‐edge was carried out for the synthesized Zn‐compound samples. The detailed instrument setup was described in the Experimental Section. XPS, FTIR, and Raman spectroscopy were also used. The structure and chemical bonding information acquired through XANES, XPS, FTIR, and Raman spectroscopy would be used for further analysis of the amperometric sensing of Gox.

**Figure 3:**
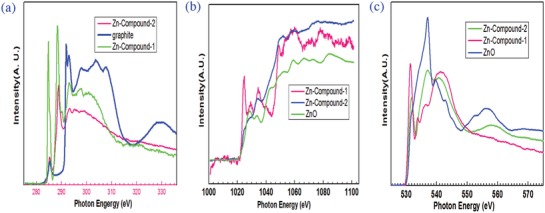
a) Carbon K‐edge XANES spectra of graphite and Zn‐Compound‐1 and Zn‐Compound‐2. b) Zinc K‐edge XANES spectra of Zn‐Compound‐1 and Zn‐Compound‐2. c) Oxygen K‐edge XANES spectra of Zn‐Compound‐1 and Zn‐Compound‐2.

**Figure** **3**a shows the carbon K‐edge XANES spectrum of a graphite foil with the absorption spectra of Zn‐Compound‐1 and Zn‐Compound‐2 samples. Two distinctive peaks located at 285.4 and 292.0 eV in the graphite foil spectrum are attributed to the transitions from carbon 1s to sp^2^ derived (C=C) π* and σ* unoccupied bands, respectively. Zn‐Compound‐1 and Zn‐Compound‐2 show similar trend to the graphite foil in the carbon K‐edge. However, comparing the carbon K‐edge XANES spectra of the graphite foil with Zn‐Compound‐1 and Zn‐Compound‐2, we observed a significant resonant peak around 288.0 eV existing for Zn‐Compound‐1 and Zn‐Compound‐2. This feature is associated with to the resonance from C=O bond. Additionally, carbon K‐edge XANES spectra of Zn‐Compound‐1 show peaks at 285.0, 288.4, 293.1, and 297.0 eV and a shoulder at 301.0 eV. The peaks at 285.0, 288.4, and 293.1 eV are associated with C=C 1π*, C=O, and C—C σ*.^23^, ^24^ The peak at 297.0 eV and the shoulder at 301.0 eV are possibly due to the resonance of C—H—O groups.^25^ Carbon K‐edge XANES spectra of Zn‐Compound‐2 show peaks at 285.5, 288.8, 293.2, and 295.0 eV and a shoulder at 300.0 eV. The peaks at 285.5, 288.8, and 293.2 eV are assigned to C=C 1π*, C=O, and C—C σ*.^23^, ^24^ The peak at 295.0 eV and the shoulder at 300.0 eV are considered to be the resonance of C—O and C=O groups.^25^

ZnO powder was used as reference material for zinc L‐edge and oxygen K‐edge XANES spectroscopy study of Zn‐Compound‐1 and Zn‐Compound‐2. Zinc XANES L‐edge spectra of ZnO powder, Zn‐Compound‐1, and Zn‐Compound‐2 samples are presented in Figure 3b. Zinc has +2 oxidation state in Zn‐Compound‐1 and Zn‐Compound‐2 samples as the absorption edges of the samples appear at the same absorption energy for ZnO powder. The feature at 1024.0 eV in the L‐edge XANES spectrum of ZnO powder is assigned to the transition from 2p to 4s derived Zn states. The other two prominent peaks, 1029.0 and 1034.0 eV are attributed to transitions from 2p electron states to 3d unoccupied states. Comparing the zinc L‐edge XANES spectrum of ZnO powder with the XANES spectra of Zn‐Compound‐1 and Zn‐Compound‐2, we notice that the intensity of the peak centered at 1024.0 eV is much higher in the spectrum of Zn‐Compound‐1 than those spectra of Zn‐Compound‐2 and ZnO. The enhancement of the intensity of a feature in XANES spectrum indicates that the density of unoccupied states increases. Since Zn 4s states are empty in ZnO, the increase in the intensity of the peak presumably is due to enhancement of the coupling and hybridization between oxygen 2p and zinc 4s states. Also, it should be mentioned that Zn‐Compound‐1, Zn‐Compound‐2, and ZnO show similar trend in the zinc L‐edge XANES spectra.

**Figure 4:**
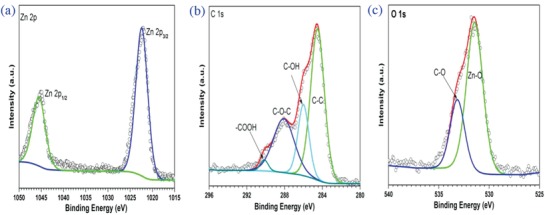
a) XPS spectra corresponding to Zn 2p of Zn‐Compound‐1. b) XPS spectra corresponding to C 1s of Zn‐Compound‐1. c) XPS spectra corresponding to O 1s of Zn‐Compound‐1.

**Figure 5:**
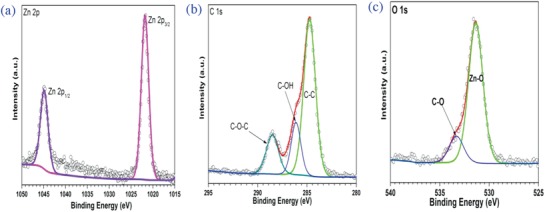
a) XPS spectra corresponding to Zn 2p of Zn‐Compound‐2. b) XPS spectra corresponding to C 1s of Zn‐Compound‐2. c) XPS spectra corresponding to O 1s of Zn‐Compound‐2.

The oxygen K‐edge spectrum of ZnO powder was used for energy calibration as well as for the identification of features in XANES spectra of Zn‐Compound‐1 and Zn‐Compound‐2 samples. Both Zn‐Compound‐1 and Zn‐Compound‐2 samples show similar XANES features in the absorption spectra plotted in **Figures** **4** and **5**.

As shown in Figure 5a, The XPS spectra of Zn 2p_3/2_ for Zn‐Compound‐2 is located at 1021.6 eV, which is assigned to Zn—O bonds.^27^ The XPS spectra of Zn 2p_1/2_ for Zn‐Compound‐2 is located at 1044.7 eV, which is similar to previous report.^27^ The XPS spectra of C 1s core level for Zn‐Compound‐2 (Figure 5b) were deconvolved into three bands located at 284.75, 286.11, and 288.49 eV, which result from the C—C bonds, C—OH bonds, and C—O—C bonds.^29^, ^30^

The peaks at 531.0 and 541.0 eV originating from π* and σ* resonances are from oxygen double bonded to carbon (C=O). The absorption features between these two resonances and residing at 534.0 and 537.0 eV are attributed to transition from hydroxyl‐derived states (O—H) and single bonded oxygen (C—O and C—O—C), respectively.^26^

Using an X‐ray photoelectron spectrometer, the states of atoms on the sample surface were analyzed. The XPS spectra of Zn 2p, O 1s, and C 1s for Zn‐Compound‐1 and Zn‐Compound‐2 were obtained and plotted in Figures 4 and 5.

As presented in Figure 4a, the XPS spectra of Zn 2p_3/2_ for Zn‐Compound‐1 is located at 1022.4 eV, which is attributed to Zn—O bonds.^27^ The XPS spectra of Zn 2p_1/2_ 28 for Zn‐Compound‐1 is located at 1045.5 eV. Here, the XPS spectra of C 1s core level for Zn‐Compound‐1 were deconvolved into four components by an interactive least‐square computer program. The peaks located at 284.45, 286.02, 288.06, and 290.32 eV (Figure 4b) are attributed to the C—C bonds, C—OH bonds, C—O—C bonds, and —COOH group.^29^, ^30^

The XPS spectra of O 1s for Zn‐Compound‐1 and Zn‐Compound‐2 (Figures 4c and 5c) show similar trend. The main peak at 531 eV is attributed to Zn—O bonding.^31^ The small peak at 533–534 eV results from C=O, C—O—C, and C—O—N species.^32^

**Figure 6:**
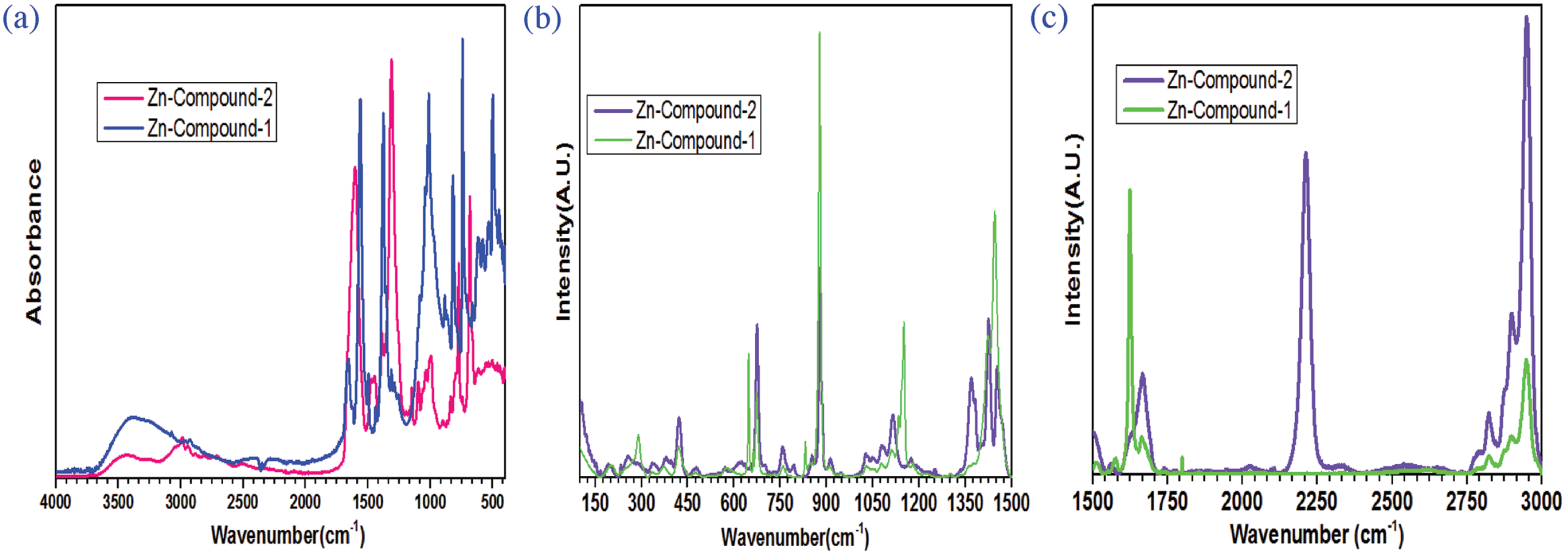
a) FTIR spectra of Zn‐Compound‐1 and Zn‐Compound‐2 samples. b) Raman spectra of Zn‐Compound‐1 and Zn‐Compound‐2 in the 100–1500 cm^−1^ region. c) Raman spectra of Zn‐Compound‐1 and Zn‐Compound‐2 in the 1500–3000 cm^−1^ region.

The FTIR and Raman spectroscopy were applied to gain more information about the chemical bonding of the Zn‐compound samples. As presented in **Figure** **6**a, the FTIR spectra of Zn‐Compound‐2 samples show the peaks at 506, 679, 772, 835, 853, 898, 1004, 1058, 1093, 1147, 1307, 1387, 1448, 1609, 2499, 2722, 2954, and 2998 cm^−1^. The shoulder band at the wavenumber of 3176–3693 cm^−1^ corresponds to stretching originated from O—H groups.^33^ The peak of 506 cm^−1^ is assigned to Zn—O bond.^32^ The peak of 679 cm^−1^ is attributed to C=C=O bending.^34^ The peaks of 835 and 898 cm^−1^ result from C—H bending.^34^ The peak of 1004 cm^−1^ is assigned to the mode contributed by the combination of C—C—C bending and C—O—H bending.^34^ The peak of 1147 cm^−1^ is considered to be the mode contributed by C—C—H bending and C=C vibration.^34^ The peak of 1609 cm^−1^ is assigned to the mode contributed by the C=C vibration and the C—C—H bending.^34^ The peaks of 2954 and 2998 cm^−1^ result from the O—H stretching.^34^, ^35^, ^36^ The peak of 853 cm^−1^ is due to the combined contribution of C—H bending and C—C—C bending.^31^ The peak of 1448 cm^−1^ is attributed to C—H stretching.^37^, ^38^, ^39^

The FTIR spectra of Zn‐Compound‐1 sample (Figure 6a) show the peaks at 451, 496, 528, 579, 612, 660, 743, 821, 876, 1010, 1310, 1384, 1438, 1499, 1557, 1661, and 3373 cm^−1^. The Zn—O bond is considered in the range of 400–600 cm^−1^.^33^ Therefore, the peaks of 451, 496, 528, and 579 cm^−1^ in the spectra is assigned to Zn—O bond. The shoulder band at around the peak of 3373 cm^−1^ is assigned to stretching of O—H groups.^1^ The peak of 821 cm^−1^ is attributed to C=C=C bending.^34^ The peak of 1384 cm^−1^ is originated from the mode contributed by the C=C vibration and C=C—C bending.^34^ The peak of 1438 cm^−1^ is assigned to CH_3_‐stretching modes.^40^ The peak of 1557 cm^−1^ is attributed to carboxylate group.^38^ The peak of 1661 cm^−1^ results from C=O vibration.^31^ The peak of 1310 cm^−1^ is attributed to C—H bending.^41^, ^42^

The Raman spectra of Zn‐Compound‐1 samples (Figure 6b,c) show the Raman bands at 195, 288, 338, 368, 421, 478, 581, 646, 676, 761, 831, 876, 909, 954, 1029, 1074, 1112, 1134, 1149, 1187, 1304, 1420, 1447, 1516, 1581, 1625, 1661, 2600, 2828, 2879, 2898, and 2946 cm^−1^. In the low frequency region, the Raman band at 195 cm^−1^ is attributed to O—Zn—O bonding.^43^ The Raman band found at 288 cm^−1^ is assigned to the vibration mode of C—COOH stretching.^28^

The peak at 581 cm^−1^ is due to the vibration of Zn—O bond.^41^ The peak at 1187 cm^−1^ is assigned to the mixture of C—O vibration and C—C=O bending.^34^ The Raman band at 954 cm^−1^ results from C—C stretching.^43^ The peak at 1149 cm^−1^ is assigned to O—O stretching.^44^ The Raman bands around 1149 cm^−1^ (1112, 1134, and 1187 cm^−1^) are generally considered due to the splittings of O—O stretching vibration.^44^ The peak at 1447 cm^−1^ results from C—H bending.^41^ The peaks of 2828, 2879, 2898, and 2946 cm^−1^ are assigned to C—H bending.^26^ The Raman band at 1516 cm^−1^ is attributed to C=C vibration.^45^ The Raman band at 1661 cm^−1^ results from C=O vibration.^46^ The Raman bands at 909 and 954 cm^−1^ are assigned to C—H bending.^46^

The Raman spectra of Zn‐Compound‐2 samples (Figure 6b,c) show the Raman bands at 195, 231, 255, 336, 377, 397, 421, 480, 576, 624, 679, 699, 756, 792, 854, 877, 911, 1029, 1049, 1082, 1114, 1174, 1368, 1422, 1457, 1506, 1562, 1669, 2026, 2031, 2106, 2211, 2337, 2547, 2651, 2823, 2900, and 2954 cm^−1^. The peak at 195 cm^−1^ is attributed to O—Zn—O bonding.^43^ The peak at 576 cm^−1^ is due to the vibration of Zn—O bond.^41^

The Raman bands at 911, 1049, 1114, 1368, 1422, 1457, 2823, 2900, and 2954 cm^−1^ result from C—H bending.^46^ The Raman band at 1669 cm^−1^ is due to the mixture of C=O vibration and C=C vibration.^46^ The peak at 1174 cm^−1^ is attributed to O—O stretching.^44^

**Figure 7:**
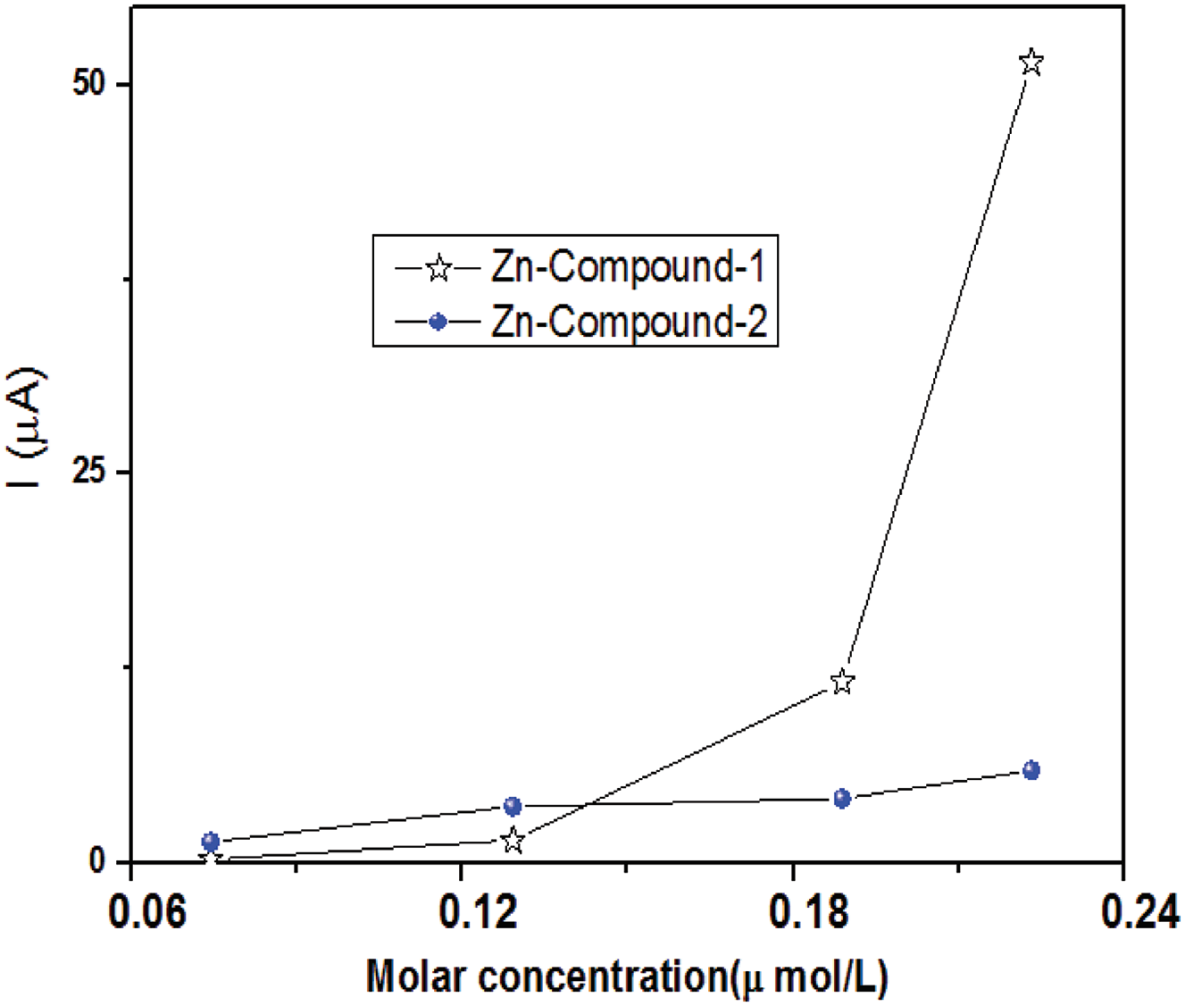
The current generated is increasing when the molar concentration of glucose oxidase is increasing.

**Figure** **7** indicates the sensing of glucose oxidase using Zn‐Compound‐1 and Zn‐Compound‐2. The schematic of the measurement circuit is shown in Figure 2c. The wired amperemeter shows current increases when glucose oxidase is added to the silicone foam by a pipette. In order to explain this phenomenon, we started from the analysis of the defect states in Zn‐compound samples. We consider one possible mechanism is due to the formation of extra bands along with the defect states. A detailed discussion is given in the following.

As derived from Tables S2 and S3 in the Supporting Information (see the XPS survey results in Section SII of Supporting Information), the N 1s shows lower value of peak areas and atomic number comparing to that of C 1s, O 1s and Zn 2p, which indicates that N 1s exists in the defect sites.

The small amount of N 1s could be originated from the organic reactant. They can combine with the small amount of the remaining starting materials to form a complex structure. That is undetectable in the Raman/XANES spectra due to the resolution limitation of the instruments or appears as small peaks in the spectra profile.

This is consistent with our finding of the Raman/FTIR/XANES/XPS spectra. As listed in the paragraph describing the Raman spectra, there are some peaks unassigned, such as the Raman bands at 338, 368, 421, 478, 646, 676, 761, 831, 876, 1029, 1074, 1304, 1420, 1581, 1625, and 2600 cm^−1^ for Zn‐Compound‐1 and the Raman bands at 231, 255, 336, 377, 397, 421, 480, 624, 679, 699, 756, 792, 854, 877, 1029, 1082, 1506, 1562, 2026, 2031, 2106, 2211, 2337, 2547, and 2651 cm^−1^ for Zn‐Compound‐2. Also, as stated in the section of FTIR spectra, there are some peaks undetermined, including the peaks at 772, 1058, 1093, 1307, 1387, 2499, and 2722 cm^−1^ for Zn‐Compound‐2 and the peaks at 612, 660, 743, 876, 1010, and 1499 cm^−1^ for Zn‐Compound‐1. Those unassigned Raman/FTIR bands can be attributed to the vibration or stretch of C—H—O—N species or the Zn—N—O species or their mixing. Furthermore, as presented in the section of XANES spectra, the Zn K‐edge spectra of Zn‐Compound‐1 and Zn‐Compound‐2 show small resonant peaks in the spectra tail, which can be attributed to the defect states of the samples. Another evidence is from the measured XPS spectra for N 1s (see Figure S6, Supporting Information), which showed a peak at 403 eV. This peak is in accordance with the vibration from complex C—N structure.^47^, ^48^, ^49^, ^50^, ^51^

All the observation confirms that N‐based complex structure exists as defects. They are contributing to certain sources of the defects existing in the Zn‐compound samples. In the following, we will discuss how the defects in Zn‐compound would impact the formation of electric current for detection of Gox.

It should be mentioned that the dielectric constant for electrons around these defects would be higher.^50^ Generally, the Bohr radius is expressed as^50^

(1) $$\alpha_{H}=\frac{\hbar^{2}\kappa }{me^{2}}$$

where κ is the permittivity, *m* is the electron mass, *e* is the electron charge, and *ħ* is the Planck's constant. In this case, those electrons with a higher dielectric constant tend to occupy larger Bohr orbitals. They would introduce an extra band. When certain concentration of the Gox was added to the silicone foam filled with Zn‐compound samples, the Gox was bound to the Zn‐compound.

**Figure 8:**
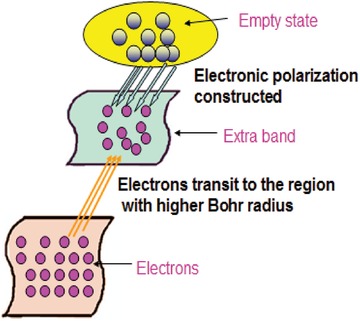
Schematic of the formation of the current. The electrons of the atoms of the Zn‐Compound‐1 or Zn‐Compound‐2 around the defect states form an extra band. Then, the interactions between these electrons and the empty states of the atoms of glucose oxidase would result in the formation of electrical polarization. Finally, electric current is produced due to the distribution of electric charges in space.

As shown in **Figure** **8**, interactions appear between the extra band and the empty state of the atoms in the sides of Gox. Therefore, electrical polarization is formed in the extra band and electrical coupling is introduced. Electrical current would be generated due to that polarization, whose magnitude is expressed by the Mott–Gurney equation

(2) $$I=\frac{9}{8}\left(\frac{A\varepsilon_{0}\varepsilon_{r}\mu P^{2}}{L}\right)$$

where *I* is the current, *A* is the effective area, ε_0_ is the vacuum permittivity, ε_r_ is the effective permittivity, *µ* is the effective permeability, *L* is the effective length, and *P* is the polarization. If more Gox is added, more electrical coupling will be introduced, leading to the formation of larger electrical polarization. This is the reason why larger electrical current was observed when larger concentration of Gox was added.

Comparing to those strategies via Raman spectroscopy /NMR/luminescent/fluorescence/absorption/ impedance spectroscopy or electrochemical station, our strategy is simpler/cheaper. This is the reason why one may use our method to estimate Gox. The well‐known Bradford method may be used to examine the concentration of enzymes. However, it has to be done via a bulky optical spectrometer. Our method is cheaper and convenient for field application, as we only need the electrical current reader/capacitor/electrical switch.

However, we admit that this Zn compound/silicone hybrid porous foam device could not be used for successive measurements. The cost of dumping the Zn‐compound/silicone hybrid foam is still high. A “green” technology should be developed. Much simpler and much more cost‐effective synthesis of metal organic compound should be developed for large‐scale application. The consideration on these impacts is valuable and paves the way for our future endeavor in practical application of amperometric sensing of glucose oxidase using the Zn‐compound filled silicone foam.

## 3 Conclusions

We present an example of fabricating Zn‐compound/silicone hybrid foams. Here, two Zn‐compounds were synthesized through a room temperature route while silicone foam was made via a facile solid‐filling‐melting technology. XANES measurements of the carbon and oxygen K‐edge and Zinc L‐edge, XRD, XPS, FTIR, and Raman were conducted for characterization of the Zn‐compounds. Most importantly, for the first time, we demonstrated the application of the Zn‐compound/silicone hybrid foam in amperometric Gox sensing. This effect was possibly due to the extra bands formation. In term of the use of simple chemistry and facile fabrication, our technique may provide a rapid method to fabricate field detection devices for a variety of bio‐chemical sensing/analysis applications. Further studies on related metal compounds in 3D porous matrix and their applications in highly selective bio‐chemical sensing are in progress.

## 4 Experimental Section

*Materials*: The Raw materials used include glucose oxidase (Sigma), zinc acetate dihydrate (Alfa Aesar), acetylenedicarboxylic acid (Alfa Aesar), terephthalic acid (Alfa Aesar), triethylamine (Alfa Aesar), *N*,*N*‐dimethylformamide (Alfa Aesar), 200 Prof Pure ethanol (Koptec) silicone general electric, and sodium sulfate hydrate (Alfa Aesar).

*Synthesis*: A brief description of the synthesis of Zn‐Compound‐1 and Zn‐Compound‐2 is given as following. For synthesis of Zn‐Compound‐1, zinc acetate dihydrate (17 g) was mixed with *N*,*N*‐dimethylformamide (100 mL) and stirred for 1 h. An organic solution was prepared by mixing terephthalic acid (5 g) and triethylamine (9 mL) and *N*,*N*‐dimethylformamide (50 mL). The prepared zinc solution and organic solution were mixed and stirred for 1 h to generate light yellow precipitate. The light yellow precipitate was washed by ethanol, filtered, and stored. For synthesis of Zn‐Compound‐2, zinc acetate dihydrate (8 g) was mixed with *N*,*N*‐dimethylformamide (50 mL) and stirred to get a clear solution. An organic solution was prepared by mixing acetylenedicarboxylic acid (0.8 g), *N*,*N*‐dimethylformamide (60 mL) and triethylamine (5 mL). The zinc solution and the organic solution were mixed and stirred to get a white precipitate. The stirring was continued for 1 h and the reaction was stopped. The acquired precipitate was washed by ethanol, filtered, and stored for further characterization.

*SEM, XRD, FTIR, Raman, and XPS Characterization*: The morphology of the silicone foams filled with Zn‐compound were studied via electron microscopy. SEM was conducted using an FEI Quanta 200 SEM.

ThermoFisher Nicolet IS 10 Fourier‐transform infrared (FTIR) spectrometer equipped with Smart iTR diamond attenuated total reflectance (ATR) accessory was used to collect the FTIR spectra of the samples. FTIR spectra collected with deuterated‐triglycine sulfate (DTGS) detector were averaged of 32 scans with 4 cm^−1^ resolution in the frequency range of 4000–400 cm^−1^.

Raman spectroscopy was done via a Johin Yvon Horiba LABRAM Integrated Raman Spectroscopy System with a HeNe laser (excitation wavelength: 632.81 nm). Raman spectra were acquired in the wavenumber range of 100–3000 cm^−1^ with a scanning step of 0.2 cm^−1^.

XRD spectra were collected with Cu *K*_a_ radiation of 0.15406 nm. The simulation of XRD profile was done through a GSAS software package. The structure of Zn‐Compound‐1 and Zn‐Compound‐2 was also solved through using GSAS. The crystal structure was drawn by using DRAWxtl software package.

XPS analyses were performed via a K‐a XPS instrument (Model: Kratos AXIS 165, Kratos Analytical, USA).

*Soft X‐Ray Absorption Spectroscopy Near Edge Structure (XANES) Measurements of the Carbon and Oxygen K‐Edge and Zinc L‐Edge*: XANES measurements of the carbon and oxygen K‐edge and Zinc L‐edge were undertaken at the variable‐line‐space plane‐grating‐monochromator (VLSPGM) beamline of the Center for Advanced Microstructures and Devices synchrotron facility at Louisiana State University, USA.

The beamline that provides a resolution better than 0.2 eV at the carbon K‐edge was shown in detail in ref. 51. The VLSPGM beamline monochromator delivers monochromatic photons at the energy range of 180–1200 eV with interchangeable two gratings.

All measurements were performed in total electron yield technique by measuring the sample's drain current with 0.2 eV energy step size. The normalization of the spectra was acquired by monitoring the primary beam current on the gold sputtered mesh located in front of the incident beam just before the sample chamber. The base pressure of the chamber was kept at 2e‐9 Torr. The energy scale of the gratings were calibrated using graphite for the carbon K‐edge and ZnO powder for the oxygen K‐edge and Zinc L‐edge. Films of Zn‐Compound‐1 and Zn‐Compound‐2 were prepared for soft X‐ray absorption measurements by placing a couple of drops of ethanol diluted samples onto gold‐coated silicon wafers.

*Electrical Current Measurement*: Figure 2c shows the setup of electrical current measurement for sensing glucose oxidase with different concentration. The silicone foam was filled with Zn‐compound samples. An electrical switch was in parallel connection with the foam and a commercial capacitor (capacitance: 10 µF). The silicone foam was filled with Zn‐compound samples. Silver paste was used for electrical contact. After glucose oxidase was injected into the foam by a syringe, electrical current was generated and stored in a capacitor. Here, the switch was first open when the current was generated. After 40 s, the switch turned closed, the capacitor discharged through a microampere meter and the current was measured. It should be mentioned the amperometric sensing ability of Zn‐compound/silicone foam was also tested by using the solution of bovine serum albumin protein or water. However, no electrical current was generated. Glucose oxidase was the only active medium which was found so far that can generate current when it was interacted with the Zn‐compound filled foam.

## Conflict of Interest

The authors declare no conflict of interest.

## Supporting information

Supplementary
