# Supplementary material for: 3D Macroporous Zinc Compound/Silicone Hybrid Foams for Amperometric Sensing of Glucose Oxidase
Source: Glob Chall. 2018 Nov 25;3(2):1800049. doi: 10.1002/gch2.201800049 (PMC6607124; doi:10.1002/gch2.201800049)
Supplement: Supplementary file 1 — Supplementary [file GCH2-3-na-s001.pdf]

Copyright WILEY-VCH Verlag GmbH & Co. KGaA, 69469 Weinheim, Germany, 2018.

Supporting Information

for *Global Challenges,* DOI: 10.1002/gch2.201800049

3D Macroporous Zinc Compound/Silicone Hybrid Foams for Amperometric Sensing of Glucose Oxidase

Ye Wu, Hao Fu, Weiwei Xie, Yingcheng Lin, Orhan Kizilkaya,* and Jian Xu**

Supplement information:

Section I: The experimental XRD spectra of Zn-Compound-1 **and Zn-compound 2 are shown in Fig. S1.**

a)

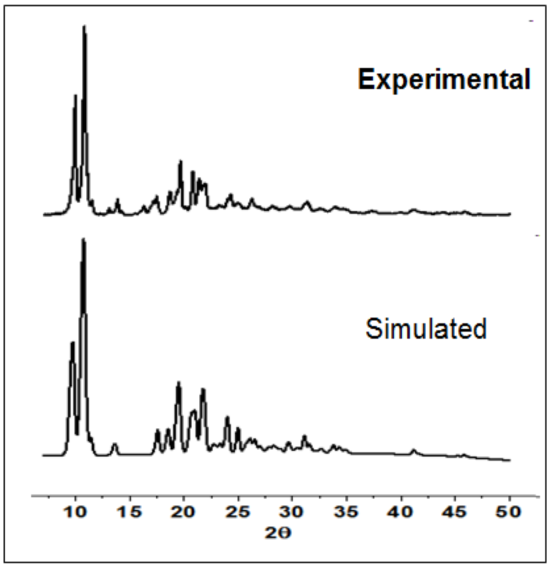

b)

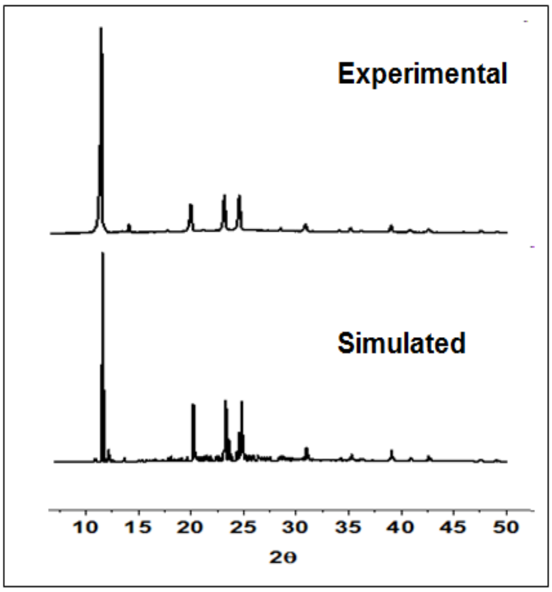

Fig.S1 (a) The experimental and simulated XRD profile of Zn-Compound-1. (b) The experimental and simulated XRD profile of Zn-Compound-2.

a)

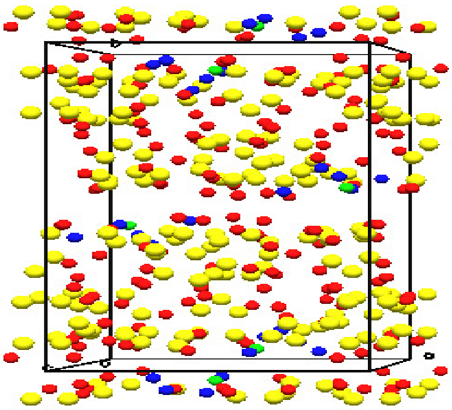

b)

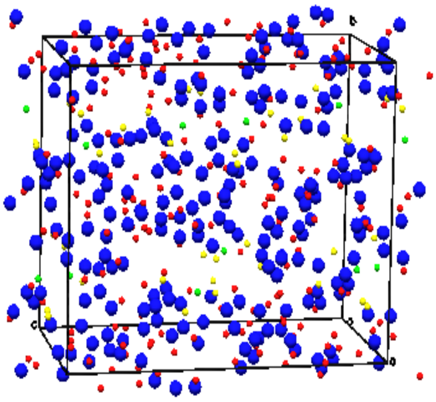

Fig.S2 (a) The crystal structure of Zn-Compound-1.(Red sphere: H atoms; Green sphere: Zn atoms; Blue sphere: O atoms; Yellow sphere: C atoms.)(b) The crystal structure of Zn-Compound-2. (Red sphere: H atoms; Green sphere: Zn atoms; Blue sphere; C atoms; Yellow sphere: O atoms.)

	Zn-Compound-1	Zn-Compound-2
Cell length	a =29.7 Å;<br>b = 9.7Å;<br>c = 18.0 Å.	a=11.7078 Å;<br>b = 18.5753Å<br>c = 11.9347Å
Cell angle:	α=90.0;<br>β=90;γ=90.0	α=90.0;<br>β=99.7;γ=90.0
Space group	Aba2	P 21/n
symmetry	orthorhombic	monoclinic

Table S1 Crystal cell parameters of Zn-Compound-1 and Zn-Compound-2.

Crystallographic data (excluding structure factors) for the structures of Zn-Compound-1 and Zn-Compound-2 reported in this manuscript have been deposited with the Cambridge Crystallographic Data Centre as supplementary publication no. CCDC- 1844584 and CCDC-1844585...

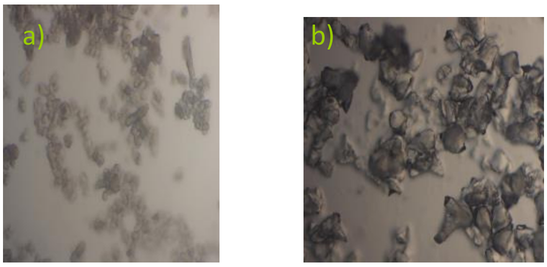

Fig. S3 (a) Optical microscopy image of Zn-Compound-1 powder. (b) Optical microscopy image of Zn-Compound-2 powder.

Section II: XPS result of survey.

Elemental ID and Quantification

Name	Peak BE	FWHM eV	Area (P) CPS.eV	Atomic %	Q
Zn2p	1023.91	4.10	91294.76	7.48	1
O1s	533.17	3.39	45314.56	24.29	1
C1s	286.46	2.68	47529.91	61.60	1
N1s	403.61	2.11	7925.07	6.63	1

Table S2. XPS survey result list of Zn-Compound-1

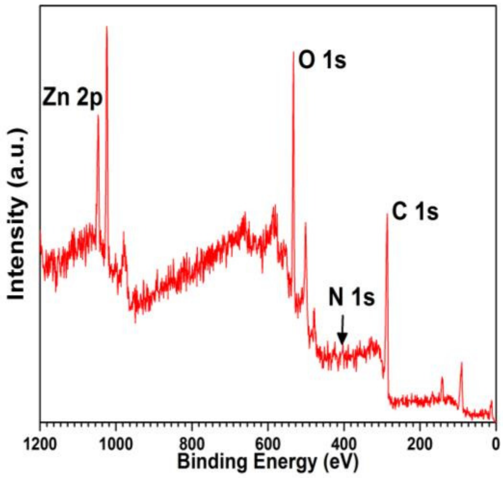

Fig. S4. XPS survey of Zn-Compound -1.

Elemental ID and Quantification

Name	Peak BE	FWHM eV	Area (P) CPS.eV	Atomic %	Q
O1s	532.43	3.06	41747.24	26.18	1
C1s	286.16	2.97	41924.06	63.57	1
Zn2p3	1022.49	1.58	27241.74	3.88	1
N1s	402.88	3.73	6514.38	6.37	1

Table S3. XPS survey result list of Zn-Compound-2.

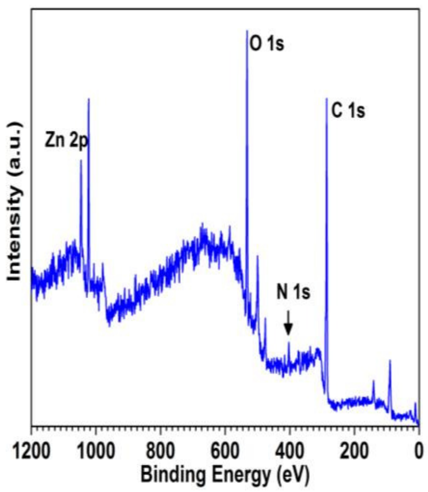

Fig. S5. XPS survey of Zn-Compound-2.

Section III: XPS spectra of N 1s for Zn-Compound-1 and Zn-Compound-2

a)

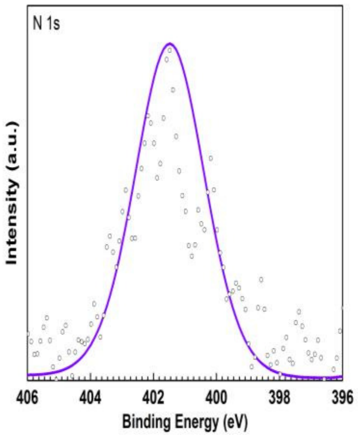

b)

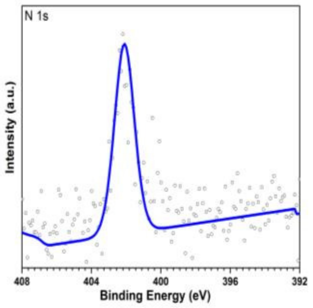

Fig. S6. (a) XPS spectra of N 1s for Zn-Compound-1. (b) XPS spectra of N 1s for Zn-Compound-2.
